# The Homœopathic Medical Council

**Published:** 1891-08

**Authors:** E. A. Krusen

**Affiliations:** Secretary


### THE HOMŒOPATHIC MEDICAL COUNCIL.

The regular meeting of the Homœopathic Medical Council was held May 20th, at the Phœnixville Club-house, Phœnixville, Pa. The following members were present: Drs. R. Farley, M. Preston, L. Hoopes, W. M. James, W. A. D. Pierce, H. Wright, E. A. Krusen, and Dr. Adair, of New York.

The meeting was called to order by the President, Dr. R. Farley.

First case was reported by Drs. Farley and Wright. Mrs. I. J. T., æt. about thirty-five years, dark complexion, tall, and finely built, mother of one child. Diagnosis, scirrhus of the stomach. Saw her in consultation April 15th, 1891. Had been under the care of allopathic physicians, and they proposed an operation as her only chance, telling her friends she had a malignant growth either of the uterus or stomach. I cannot imagine why they could not locate it, as I found it very distinctly in the epigastrium, a hard, irregular mass about the size of an egg. Her allopathic physicians said she needed an operation. Pelvic adhesions, which they said existed, caused her condition of alternate constipation and diarrhœa.

Examination for the remedy elicited the following symptoms: stools, yellow, brown, involuntary, offensive, putrid; frequently fifteen to twenty stools per diem, accompanied by much noisy flatus.

Paresis, beginning in the feet and ascending to arms. When I saw her her limbs were powerless except slight power in forearms. Frequent exclamations of "I am so tired," very restless, with desire to be moved continually.

Urine and stool passed together, but can control the urine. The involuntary stool occurred each time she was moved and when she took a drink. Gurgling in abdomen during and after drinking.

Periodical occurrence of sticky, albuminous leucorrhœa. Throat and mouth dry as a board, lips stick to teeth and gums, licking lips continually in efforts to moisten them. Thirst for large draughts of cold water, followed by vomiting. Almost constant vomiting with terrible retching, better from hot water.

Sleepless, cannot stop her thinking, many ridiculous thoughts crowd through her mind; weak attacks, with coldness, both objective and subjective, and sensation as if sinking, sinking down to the cellar.

Burning of feet, sticks them from under covers. Sensation of heat, can hardly bear sheet on her; eructations copious, noisy, and bitter.

Regurgitation of bile, saliva, mucus, food, and water; crampy pains in thighs, popliteal space, and legs, with muscular twitching during the paroxysms of pain.

Taking the case exhausted her.

℞ Phos.$^{2c}$ one dose dry.

Eight hours later her physician reports her as better; can put her limbs just where she chooses. She improved rapidly until April 21st—that is, for six days—then she began to grow worse, and her physician gave her two or three doses of Phos.$^{2c}$ and she continued to grow worse. I saw her again on the 23d, and took the case again as follows: Lacerating pain in the urethra precedes urination. Involuntary urination without sensation (Bry.), only knows when it has occurred when her clothes feel wet.

Lies supine with knees drawn up to abdomen. Rumbling and gurgling in abdomen, weakness and sinking and restlessness same as before. Hopeless of recovery.

Extreme soreness of the flesh, dreads to be touched, says bones are coming through the skin, worse from motion.

Paresis has not returned, but is too weak to move; has to be handled like an infant.

Aching pain in forehead, worse in afternoon. Aching in nape and occiput, better from cold applications; stools mushy and brown, yellow, watery, and undigested, about ten stools per diem; vomiting, retching, regurgitation, and eructations continue. Throat feels dry and scalded after emesis. Feet cold.

Heat and burning of spine.

Weak attacks last from about four P. M. until five A. M.; better for vomiting.

Craves soda water and oysters, the latter taken with benefit, the former not allowed. Nausea precedes and accompanies the emesis. Soreness and dread of being touched is only during the weak attacks, with cold feet to knees. Feet hot and dry at times, must uncover them. Crampy pains are now confined to the calves.

Flatus with stool is quiet.

Pain, and desire for stool, in abdomen before stool; better after stool.

Pulse, 112; temperature, 100°–102°.

Emaciation, sunken cheeks and eyes, brown fur on tongue, irritability, ravenous hunger with sense of emptiness in stomach, milk disagrees. Marked pulsation in the epigastrium. The case still called for Phos., so in spite of the gravity of the case, and, too, because of the extreme danger, I advised our waiting another day for the reaction of the vital energy, and it was only the third day since the repetition of the Phos., and this remedy seems to be disposed to frequently get in its work on the third day. We therefore gave the second-best remedy in the materia medica, and anxiously awaited the morrow, and when the morrow came her physician reported her "away up," and thought one physician would be enough for her for the present. She has steadily improved, and now enjoys a carriage ride daily, and not more than two (normal) stools per day. She vomits no more and eats heartily, says she has been made all over again. I will report the "malignant growth" later, unless Dame Nature hides it so I cannot find it.

CASE NO. 2, reported by Dr. Farley. Herbert W., æt. four years, scarlatina and rheumatic fever. About the seventh day of scarlatina the rheumatism began attacking joints of all the limbs and changing *repeatedly from side to side* and from legs to arms; the wrists and ankles were principally involved. Rapid grunting respiration, picks the nose and lips, the latter are dry and scaly.

Paroxysms of incessant hacking cough, better by eructations. Pain in cardiac region and stomach, better by eructations. Two or three musty yellow stools per diem.

Urine scanty and concentrated.

Restlessness, profuse perspiration, occasionally can lie on either side or supine, with head high. Hands have an œdematous appearance, are not so in joints. Joints hot and pale, restless but dreads motion, suddenly it left joints and attacked heart, producing the pain and marked murmur with first sound of heart, heard at apex (this followed the administration of Lac-can.), complains of being tired, much better from Kalmia.

On motion, the business of the meeting was suspended until after dinner, when Dr. Wright produced a patient, male, æt. sixty, affected with two corneal tumors of left eye, dark purplish in appearance, freely movable, fed by very much enlarged blood-vessels of the conjunctiva, pain in left eye running back to occipital protuberance of same side. Pains in eye better by the application of hot water. Reading causes smarting of the eye. Burning pains instantly better by hot water. No specific history could be elicited. Remedies suggested were Ars-alb. and Comocladia.

Dr. Preston then reported the two following cases:

CASE 1.—Patient æt. sixteen, slim, tall, fine haired, and of a contrary, complaining disposition. Paralysis of left lower extremities with soreness of the anterior part of the thigh, and of the popliteal space. Inability to turn in bed. Lying on face or back the preferable position when sleeping. Inability to bear the least weight on that side when walking, can draw the limb backward, but cannot extend the thigh in the least. Phos., Mag-carb., Plumb. have produced amelioration but no real improvement in strength of limb.

CASE 2.—Patient male, æt. seventy-one years, jaundiced and quite yellow over entire body and conjunctiva. Stools white, chalky white, small as if squeezed through a narrow place in the bowels. Every few days there is a cold spell and chill followed by heat and thirst, restless nights with smothering spells and inability to lie in bed. The thirst precedes the coldness, which is also preceded by drowsiness, heat is followed by sweat, which gives relief. Urine is dark and contains bile, almost normal in quantity. Patient has no appetite on account of dry, sticky tongue; takes only milk and soup; can't chew or swallow because bolus becomes too dry. He hawks phlegm and belches fluid after eating or drinking, craves beer and wine. Has taken Myrica., Card-mar., Cancer-flu.

A suggestion as to the proper remedy is desired.

During the meeting there were some interesting discussions of which the following are the most valuable points:

Kalmia has pains running from the hips down to the feet, or from the knees down to the feet.

RHUS-TOX POISONING.—Dr. Hoopes, of West Chester, related a case of crusta lactea complicated with erysipelas occurring in a child, which came under his care. By advice of another physician he gave Rhus-tox.$^{cm}$ in water, a teaspoonful every three hours, for about twenty-four hours. Upon his next visit to the case the mother of the child called him to account for "poisoning" her child with Rhus-tox. He asked her how she knew that he had given Rhus-tox. The mother answered that she could not be deceived, for she had witnessed too many cases of Rhus poisoning not to know it when she saw it.

Dr. Mahlon Preston, of Norristown, said that the best antidote for Rhus-tox. poisoning is Apis$^{85m}$, Jenichen. He has had cases where the eruption had assumed the vesicular character; the vesicles being very large. The effect of the Apis in such cases is miraculous. Euphorbium is also a remedy that must not be forgotten in cases of Rhus poisoning, especially when the vesicles are very large.

Dr. Hoopes had given Bryonia in cases of Rhus-tox. poisoning with excellent effect. He related the case of an old man who was a great skeptic in regard to Homœopathy, who was suffering from Rhus poisoning. The doctor meeting him accidentally offered him a dose of Bryonia$^{2c}$, a single powder. The sufferer agreed to take it, though avowing his disbelief in its efficacy. Within an hour the itching ceased and the patient speedily got well, to his great surprise.

Another case was that of a boy who went with other children to bathe in a small creek. Whilst standing naked upon the bank of the stream he was playfully pushed by another boy into a mass of poison ivy and was terribly poisoned. The itching was so intense that his mother spent the evening rubbing him. She sent for Dr. Hoopes, who gave Bryonia$^{2c}$, and the itching ceased very shortly, and the eruption got well gradually without any further irritation.

Baby Food.—Dr. Pierce said that he directs that the milk should stand for several hours until the cream rises. The cream is then to be skimmed off with the upper layer of milk and into it is put some ground sugar of milk, a teaspoonful of the sugar to a glassful of milk.

Dr. Farley said that his preparation was half a teaspoonful of milk sugar and half a teaspoonful of cane sugar to each tumblerful of milk.

Dr. Preston says, Let the milk stand for ten or twelve hours and then skim off the cream together with the upper third of the milk. Then add from one-third to one-fourth of its bulk of water and two or three grains of sugar of milk.

Dr. Preston adopts the rule of never giving solid food to babies until the teeth have grown.

Dr. Pierce never gives solid food to babies until the incisor teeth have grown. He then allows meat, as he considers that the appearance of the incisors is the indication for meat.

Speaking of neuralgic pains in the thighs, it was said that Xanthoxylum has pain upon the anterior surface of the thigh, and is therefore a useful remedy in this condition.

The Society then adjourned. E. A. KRUSEN, *Secretary.*

